# Characterization of the Immune Cell Infiltration Landscape Uncovers Prognostic and Immunogenic Characteristics in Lung Adenocarcinoma

**DOI:** 10.3389/fgene.2022.902577

**Published:** 2022-05-23

**Authors:** Xin Wang, Zhenyi Xu, Zhilin Liu, Weihao Lin, Zheng Cao, Xiaoli Feng, Yibo Gao, Jie He

**Affiliations:** ^1^ Department of Thoracic Surgery, National Cancer Center/National Clinical Research Center for Cancer/Cancer Hospital, Chinese Academy of Medical Sciences and Peking Union Medical College, Beijing, China; ^2^ Department of Epidemiology and Biostatistics, School of Public Health, Harbin Medical University, Harbin, China; ^3^ Department of Biostatistics, Peking University, Beijing, China; ^4^ Department of Pathology, National Cancer Center/National Clinical Research Center for Cancer/Cancer Hospital, Chinese Academy of Medical Sciences and Peking Union Medical College, Beijing, China; ^5^ Laboratory of Translational Medicine, National Cancer Center/National Clinical Research Center for Cancer/Cancer Hospital, Chinese Academy of Medical Sciences and Peking Union Medical College, Beijing, China; ^6^ State Key Laboratory of Molecular Oncology, National Cancer Center/National Clinical Research Center for Cancer/Cancer Hospital, Chinese Academy of Medical Sciences and Peking Union Medical College, Beijing, China; ^7^ Central Laboratory, National Cancer Center/National Clinical Research Center for Cancer/Cancer Hospital & Shenzhen Hospital, Chinese Academy of Medical Sciences and Peking Union Medical College, Shenzhen, China

**Keywords:** immune cell infiltration, prognosis, lung adenocarcinoma, tumor microenvironment, immunotherapy

## Abstract

The immune cell infiltration in TME has been reported to be associated with prognosis and immunotherapy efficiency of lung cancers. However, to date, the immune infiltrative landscape of lung adenocarcinoma (LUAD) has not been elucidated yet. Therefore, this study aimed to identify a new transcriptomic-based TME classification and develop a risk scoring system to predict the clinical outcomes of patients with LUAD. We applied “CIBERSORT” algorithm to analyze the transcriptomic data of LUAD samples and classified LUAD into four discrete subtypes according to the distinct immune cell infiltration patterns. Furthermore, we established a novel predictive tool (TMEscore) to quantify the immune infiltration patterns for each LUAD patient by principal component analysis. The TMEscore displayed as a reliable and independent prognostic biomarker for LUAD, with worse survival in TMEscrore-high patients and better survival in TMEscrore-low patients in both TCGA and other five GEO cohorts. In addition, enriched pathways and genomic alterations were also analyzed and compared in different TMEscore subgroups, and we observed that high TMEscore was significantly correlated with more aggressive molecular changes, while the low TMEscore subgroup enriched in immune active-related pathways. The TMEscore-low subtype showed overexpression of PD-1, CTLA4, and associations of other markers of sensitivity to immunotherapy, including TMB, immunophenoscore (IPS) analysis, and tumor immune dysfunction and exclusion (TIDE) algorithm. Conclusively, TMEscore is a promising and reliable biomarker to distinguish the prognosis, the molecular and immune characteristics, and the benefit from ICIs treatments in LUAD.

## Introduction

Although great advances have been achieved in both basic and clinical cancer research (Gu et al., 2020; Jiao and Yang, 2020), cancer still caused approximately 10 million of deaths in 2020 (Sung et al., 2021). With the high prevalence and poor prognosis, lung cancer is ranked as the first leading cause of cancer-related deaths worldwide, becoming a major global health problem (Miller et al., 2019; Siegel et al., 2019; Sung et al., 2021). Recently, the emergence of checkpoint blockade immunotherapy (Pardoll, 2012; Topalian et al., 2015) has significantly improved the strategies of LUAD. However, the minority of response and resistance to these treatments frequently impedes the clinical outcomes. Additionally, the effects of ICIs are not only driven by genetic and epigenetic alterations in tumor cells, but the tumor microenvironment (TME) has also been reported to be a crucial regulator in tumorigenesis (Dejima et al., 2021; Ye et al., 2022), development, metastasis (Quail and Joyce, 2013), and resistance to therapies (Ostman, 2012; Lu et al., 2020).

TME chiefly consists of multiple subpopulations of T and B lymphocytes, dendritic cells (DCs), macrophages, neutrophils, and myeloid-derived suppressor cells (MDSCs) (Belli et al., 2018). The balance between pro-tumorigenic and anti-tumor factors in the TME conducts tumor growth (Wellenstein and de Visser, 2018; Hinshaw and Shevde, 2019). Accumulating evidence has indicated the TME immune composition is generally correlated with prognosis and responsiveness to various cancer treatments. On one hand, tumor-infiltrating lymphocytes (TILs), such as CD4^+^ and CD8^+^ T cells, have been associated with longer survival and better response to immunotherapy (Kawai et al., 2008; Fridman et al., 2012). On the other hand, the tumor cells can promote a suppressive TME, which challenges anti-tumor immunity by inducing upregulation of inhibitory immune signaling, suppressive cytokine secretion, and recruitment of suppressive immune cells, such as tumor-associated macrophages (TAMs) presenting pro-tumor effects by secreting immunosuppressive cytokines, including interleukin-10 (IL-10) and transforming growth factor-β (TGF-β) (Mantovani et al., 2017), as well as immunomodulatory cells, such as myeloid-derived suppressor cells (MDSCs) (Ostrand-Rosenberg and Fenselau, 2018)and regulatory T cells (Tregs) (Shimizu et al., 2010), which are all associated with unfavorable prognosis. To be specific, focusing on cellular diversity shows that TME heterogeneity could impact clinical outcomes and provide a challenge for immunotherapy of LUAD (Wu F. et al., 2021; Nguyen et al., 2021). Therefore, investigating the effects of TME composition on the tumor cells will help us decode the regulation of the microenvironment by the tumor.

To date, the emerging predictors for immunotherapy in NSCLC are still imperfect, such as programmed death-ligand 1 (PD-L1) expression (Dempke et al., 2018) is thought to be induced by interferon-γ (IFN-γ)- mediated immune responses and tumor mutational burden (TMB) (Klein et al., 2021) is reported to determine the tumor immunogenicity. It is suggested that only reflecting the tumor cell intrinsic features but ignoring the extrinsic factor, especially TME, is attributed to inconsistencies. Thus, the characteristics of TME should be further comprehensively explored to determine effective biomarkers that precisely predict prognosis and considerably optimize personalized immunotherapy.

Progress has been recently achieved by immunotherapy, emphasizing the importance of TME in LUAD. It elucidates that TME is not the single-cell population but a complex interface among cancer cells, stroma, and infiltrating immune cells. Deeper analyses of the NSCLC TME are necessary to refine the potential application of these findings to clinical care. We applied “CIBERSORT” algorithm to analyze the transcriptomic data of 500 LUAD samples in TCGA and classified the LUAD into four discrete subtypes according to the distinct immune cell infiltration patterns. Furthermore, we established the TME scores to characterize and quantify the immune infiltration patterns for each LUAD patient based on the mRNA expression profiles. Conclusively, we investigated and validated the association between TME score and the clinical outcomes, as well as the efficacy of anti-PD- (L)1 treatment in LUAD, which can facilitate the identification of ideal candidates for personalized immunotherapeutic strategies.

## Methods

### Datasets and Preprocessing

A total of 1,518 lung adenocarcinoma (LUAD) and 59 normal tissue samples were retrieved and downloaded from the corresponding datasets, including TCGA LUAD from TCGA data portal (https://xenabrowser.net/datapages/) and GSE31210, GSE37745, GSE50081, GSE68465, and GSE13213 from the NCBI Gene Expression Omnibus (GEO, https://www.ncbi.nlm.nih.gov/geo/). The somatic mutation data (SNPs and small INDELs) were downloaded from TCGA database (MuTect2 Variant Aggregation and Masking). The raw data of the dataset from Affymetrix were processed using the RMA algorithm in the “Affy” package. The data from Agilent were downloaded with the processed version. For TCGA dataset, RNA-sequencing data (FPKM values) were transformed into transcripts per kilobase million (TPM) values, which are more similar to those resulting from microarrays and more comparable between samples (Wagner et al., 2012). The following inclusion criteria were used: *1*) histologically confirmed LUAD, *2*) simultaneously available information on mRNA expression profile data and OS, *3*) the sample tissue was collected from the primary solid tumor (“01”), and there was no duplication sample in TCGA, *4*) genes were recorded in all datasets, and *5*) genes with more than 70% of the missing value or 0 value were deleted. The remaining missing values were imputed with KNN imputation approaches. Therefore, 10,320 mRNAs were included in the analysis.

### Consensus Clustering for the Tumor Microenvironment

Distinguishing between tumor and normal tissue difference expression genes (DEGs) in TCGA was performed with “limma” (FDR <0.05 and |log2FC| > 1), which better identifies the characteristics of tumor. Furthermore, the tumor microenvironment was quantified by CIBERSORT (https://cibersort.stanford.edu/)(Newman et al., 2015), a deconvolution method for inference of tumor-infiltrating immune components from bulk tissue gene expression profiles. Tumors with qualitatively different immune cell infiltration patterns were grouped using consensus clustering (100 iterations, resample rate of 80%, and hierarchical cluster). This procedure was performed with the “ConsensusClusterPlus” R package.

### Identification of DEGs Associated With the TME Phenotype

To functionally elucidate the biological characteristics of the TME subtypes in LUAD, we employed random forest (RF), an efficient and reliable machine learning method to identify DEGs between subtypes of TME. We run RF 100 times with different seeds to find the duplicated variables with at least 80% repetition rate to further ensure the stability of variable selection.

### Generation of TMEscore

To further elucidate the comprehensive profile of TME characteristics, the construction of TME metagenes was performed as follows: first, we further screened candidate prognostic genes from DEGs. Next, a consensus clustering algorithm was employed to define the cluster of genes. Then, a principal component analysis (PCA) was performed, and principal component 1 was extracted to serve as the signature score. After obtaining the prognostic value of each gene signature score, we applied a method similar to GGI (Sotiriou et al., 2006) to define the TMEscore of each patient:

$$TME\text{score}=\sum PC1_{i}-\sum PC1_{j},$$

where i is the signature score of clusters whose Cox coefficient is positive and j is the expression of genes whose Cox coefficient is negative.

### Functional and Pathway Enrichment Analysis

To further analyze the biological significance of the genes related to TMEscore with KEGG and GO function analysis, the “clusterProfiler” R package was adopted to annotate gene patterns (Wu T. et al., 2021). The Benjamini–Hochberg procedure was used to control the false discovery rate (FDR). We set the cut-off of adj. *p*-values to 0.2 so that we could find more relevant pathways and functions based on the small number of DEGs. Gene set enrichment analysis (GSEA) illustrated the significantly different enriched pathways in the high- and low-TMEscore groups. Gene sets were downloaded from the MSigDB database of the Broad Institute (Subramanian et al., 2005) and employed the Hallmark gene sets and 1,000 permutations. An enrichment pathway between two subtypes was determined with an FDR of <0.25 and the normalized enrichment score (NES).

### Predicting the Patients’ Response to ICIs

The Cancer Immunome Atlas (https://tcia.at/) analyzed the immune landscapes and antigenomes of 20 solid tumors that were quantified by Immunophenoscore (IPS, a superior immune response molecular marker) (Charoentong et al., 2017). The IPS value, which ranged from 0 to 10, was positively correlated to tumor immunogenicity and could predict the patients’ response to immune checkpoint inhibitors (ICI treatment). Tumor Immune Dysfunction and Exclusion (TIDE, http://tide.dfci.harvard.edu/), a computational method to predict immune checkpoint blockade response, was developed by Jiang et al. (2018). TIDE uses a set of expression markers to profile two primary mechanisms of tumor immune evasion: T-cell dysfunction and T-cell exclusion. Patients with higher TIDE prediction scores represent a greater potential of tumor immune escape; therefore, TIDE could evaluate patients who are more likely to benefit from ICI. In addition, the mRNA expression of immune checkpoints was analyzed in different prognosis groups.

### Statistical Analysis

Continuous variables were summarized as mean ± SD, and categorized variables were described by frequency (n) and proportion (%). Differences among variables were tested by the Wilcoxon rank-sum test and Fisher’s exact tests. The relationship between variables was tested by Spearman rank correlation analysis. The cut-off value of TMEscore was calculated based on the correlation between the patients’ survival and the TMEscore in TCGA with the “survminer” package. Univariate and multivariate Cox regression analyses were used to assess prognostic analysis. Batch effects from non-biological technical biases were corrected using the “ComBat” algorithm of the “sva” package. The “Maftools” package was used to present the mutation landscape and identify the differential gene mutations between groups. The heatmap was produced by the R package “ComplexHeatmap.” A two-sided *p* < 0.05 was regarded as statistically significant. All data processing was performed in R 4.0.2 software.

## Results

### Landscape of Lung Adenocarcinoma TME

**FIGURE 1:**
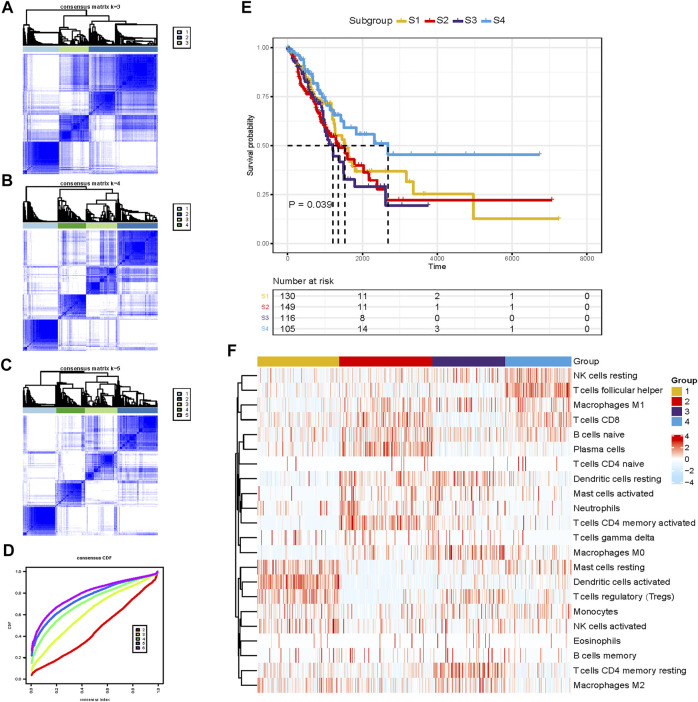
Unsupervised clustering of the tumor microenvironment (TME) cells for 500 patients in the TCGA-LUAD cohort. **(A**–**C)** Consensus matrices of different clusters. **(D)** Consensus cumulative distribution function (CDF) curve. **(E)** Kaplan–Meier (K-M) curves for overall survival (OS) of four different subtypes (log-rank test, *p =*0.039). **(F)** Abundance pattern of 22 TME cell types in four TME subtypes.

This study was conducted as per the flow chart shown in Supplementary Figure S1. The information of 1,518 LUAD patients is detailed in Supplementary Table S1. To classify the LUAD TME, the consensus clustering algorithm was used to cluster TME information obtained by CIBERSORT in TCGA-LUAD dataset (Supplementary Table S2). The most appropriate clustering number was four (Figures 1A–D), which was selected by consensus matrices and consensus cumulative distribution function (CDF) curve. This analysis revealed that LUAD can be clustered into four distinct TME subtypes termed S1–4. The patients with subtype S4 had significantly longer overall survival (OS) than patients with subtypes S1 and S2, and subtype S3 demonstrated the worst survival (Figure 1E). These four TME subtypes varied significantly based on the expression levels of LM22 gene signatures (Figure 1F). The S4 subtype was characterized by increases in the infiltration of CD 8+ T cells, resting NK cells, follicular helper T cells, and M1 macrophages, displaying S4 was significantly associated with immune activation. Meanwhile, resting mast cells, activated dendritic cells, and regulatory T cells (Tregs) were enriched in the S1 subtype, and the S2 subtype showed significant increases in the infiltration of naïve B cells, plasma cells, and CD4^+^ memory-activated T cells; on the contrary, M0 macrophages, M2 macrophages, and CD4^+^ memory-resting T cells showed high infiltration in the S3 subtype, indicating an immunosuppressive milieu. Taken together, we demonstrated that the four TME subtypes were characterized by distinct immune cell infiltration and prognosis.

### Identification of DEGs and Functional Annotation

**FIGURE 2:**
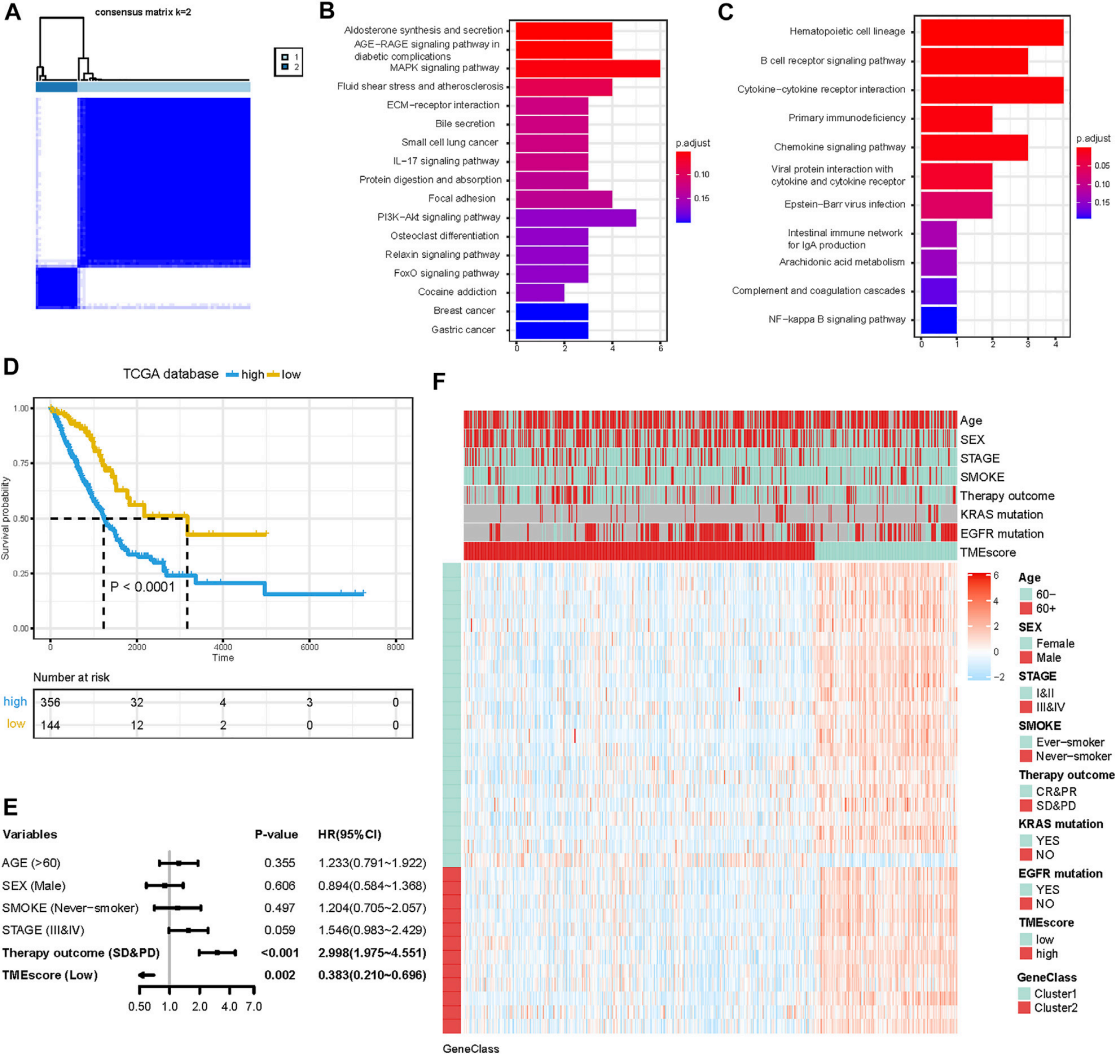
Construction of the TMEscore for LUAD patients. **(A)** Consensus matrices of differentially expressed genes (DEGs) among TME subtypes. **(B** and **C)** KEGG pathway enrichment analysis results in G1 and G2. **(D)** K-M curve for OS of different TMEscore groups (log-rank test, *p* < 0.001). **(E)** Forest plots illustrating the results of multivariate Cox proportional hazards model of clinical feature in TCGA cohort. **(F)** Heatmap of DEG expression and clinical characteristics. TMEscore, age, sex, stage, smoke, therapy outcome, mutation of KARS, and mutation of EGFR are shown as patient annotations. Gene clusters are shown as gene annotations. Top legend, gray indicates a missing value.

To further identify the biological characteristics and differences among TME subtypes, RF algorithm was employed to extract the phenotype signatures. By 100 times analysis, a total of 77 DEGs duplicated at least 80 times were identified (Supplementary Table S3). Through consensus clustering analysis based on the expression of the 77 most representative DEGs, we divided DEGs into two different clusters termed G1 (62 DEGs) and G2 (15 DEGs) (Figure 2A). These two gene clusters were closely related to distinct TME and played different biological roles. Then, GO and KEGG enrichment analyses were performed with the “clusterProfiler” R package. The G1 cluster was mainly enriched in the MAPK signaling pathway, PI3K-Akt signaling pathway, aldosterone syntheses, and focal adhesion pathways (Figure 2B). The G2 cluster was mainly enriched in hematopoietic cell lineage, B-cell receptor signaling pathway, cytokine–cytokine receptor interaction, and primary immunodeficiency (Figure 2C). Significantly enriched pathways and molecular functions are summarized in Supplementary Tables S4 and S5. Collectively, the coherence between the prognostic and biological features in the two gene subgroups indicated that this classification was reliable and reasonable.

### Construction and Validation of the TMEscore in Six Independent Cohorts

**FIGURE 3:**
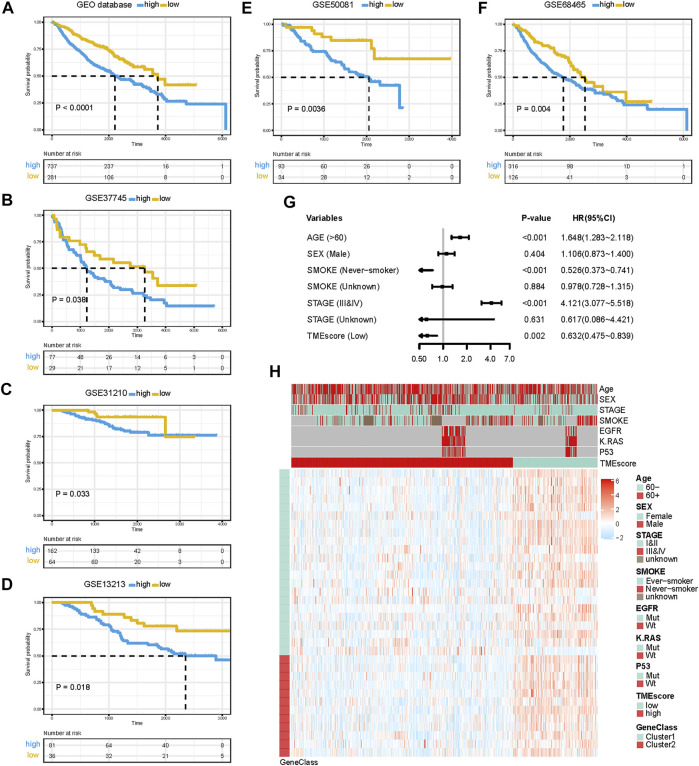
Prognostic value of TMEscore for LUAD patients in five GEO cohorts. **(A)** K-M curve of all 1,018 patients in the GEO database between different TMEscore groups (log-rank test, *p* < 0.001). **(B**–**F)** K-M curves of five independent GEO datasets in different TMEscore subgroups. **(G)** Forest plots illustrating the results of the multivariate Cox proportional hazards model of clinical features in the GEO database. **(H)** Heatmap of DEG expression and clinical characteristics. TMEscore, age, sex, stage, smoke, mutation of KARS, mutation of EGFR, and mutation of P53 are shown as patient annotations. Gene clusters are shown as gene annotations. Top legend, gray indicates a missing value.

Although the four TME subtypes were identified, their clinical significance needed to be further evaluated and quantified. Therefore, we build TMEscore based on TME information in the TCGA-LUAD cohort to assess the prognostic value. Association with a prognosis of 34 genes (G1:22, G2:12) was confirmed by Cox regression analysis. First, principal component analysis (PCA) was used to compute two aggregate scores, TMBscore A from G1 and TMBscore B from G2. Then, we performed univariate Cox regression on each TMEscore to evaluate the prognostic value. Finally, TMEscore A and TMEscore B were integrated to obtain TMEscore for each sample. The prognostic value of the TMEscore was further assessed by the log-rank test after classification as high-risk and low-risk groups based on the corresponding optimal cut-off value (−0.92) acquired by the “survminer” R package in the TCGA-LUAD cohort. We visualized gene expression and clinical features distribution in different risk groups with a heatmap in TCGA and GEO datasets, respectively (Figures 2F and 3H). The Kaplan–Meier curve of TMEscore subgroups showed that the patients in the low TMEscore group (median survival time 3,169 days) had significantly better overall survival than the high-TMEscore group (median survival time 1,235 days; log-rank test, *p* < 0.0001; Figure 2D). Moreover, the prognostic value of the TMEscore was further assessed with five external datasets in the GEO database: GSE37745, GSE31210, GSE13213, GSE50081 and GSE68465. Similar results were found that the survival advantage in the low TMEscore group in above cohorts, with the corresponding *p*-value of 0.038, 0.033, 0.018, 0.0036, and 0.004 (Figures 3B–F). Meanwhile, we integrated a total of 1,018 samples in the GEO datasets to evaluate prognostic efficiency, indicating the low-TMEscore group patients had better overall survival compared to the high-TMEscore group (log-rank test, *p* < 0.0001; Figure 3A). These findings suggested that TMEscore possessed a reliable and robust capacity for predicting the prognosis for LUAD patients.

### TMEscore Was an Independent Prognostic Factor for LUAD Patients

In addition to the TMEscore, other prognostic factors such as individual and clinicopathological features were included. After multivariable adjustments with age, sex, smoke situation, TNM stage, and therapy outcomes in the TCGA cohort, the TMEscore was confirmed as an independent prognostic indicator with a hazard ratio of 0.383 [95% CI: 0.210–0.696] in the TCGA-LUAD cohort (Figure 2E), 0.632 [95% CI: 0.475–0.839] in the GEO datasets (Figure 3G). Elder, ever-smoker, advanced stage, and non-response to therapy were also suggested to be independent risk factors in different datasets, respectively.

Recent studies have reported that specific gene alterations, such as TP53 (Sun et al., 2020), KRAS (Hamarsheh et al., 2020), EGFR (Chen et al., 2015), and STK11 (Mazzaschi et al., 2021) have an important role in the regulation of the tumor immune microenvironment (TIME) and served as biomarkers to tumor therapeutics (Lee et al., 2017; Krishnamurthy et al., 2021). We further explore the predictive value of this TMEscore in LUAD patients with EGFR/KRAS mutation (MUT) or wild type (WT). Remarkably, this risk model had predictive power for both EGFR wild type and EGFR mutation LUAD patients, except for patients with TP53/EGFR co-mutations (Supplementary Figure S2). Similarly, this risk model exhibited a robustly predictive value in both KRAS wild type and KRAS mutation LUAD patients, except for patients with KRAS/STK11 co-mutations LUAD patients (Supplementary Figure S3). Among the EGFR/KRAS wild-type/mutation population, the beneficial trends of low TMEscore in the prognosis of LUAD patients were observed in distinct subgroups, suggesting that TMEscore was an independent and reliable prognostic indicator.

### Different Biological Processes Between the High-TMEscore Group and the Low-TMEscore Group

**FIGURE 4:**
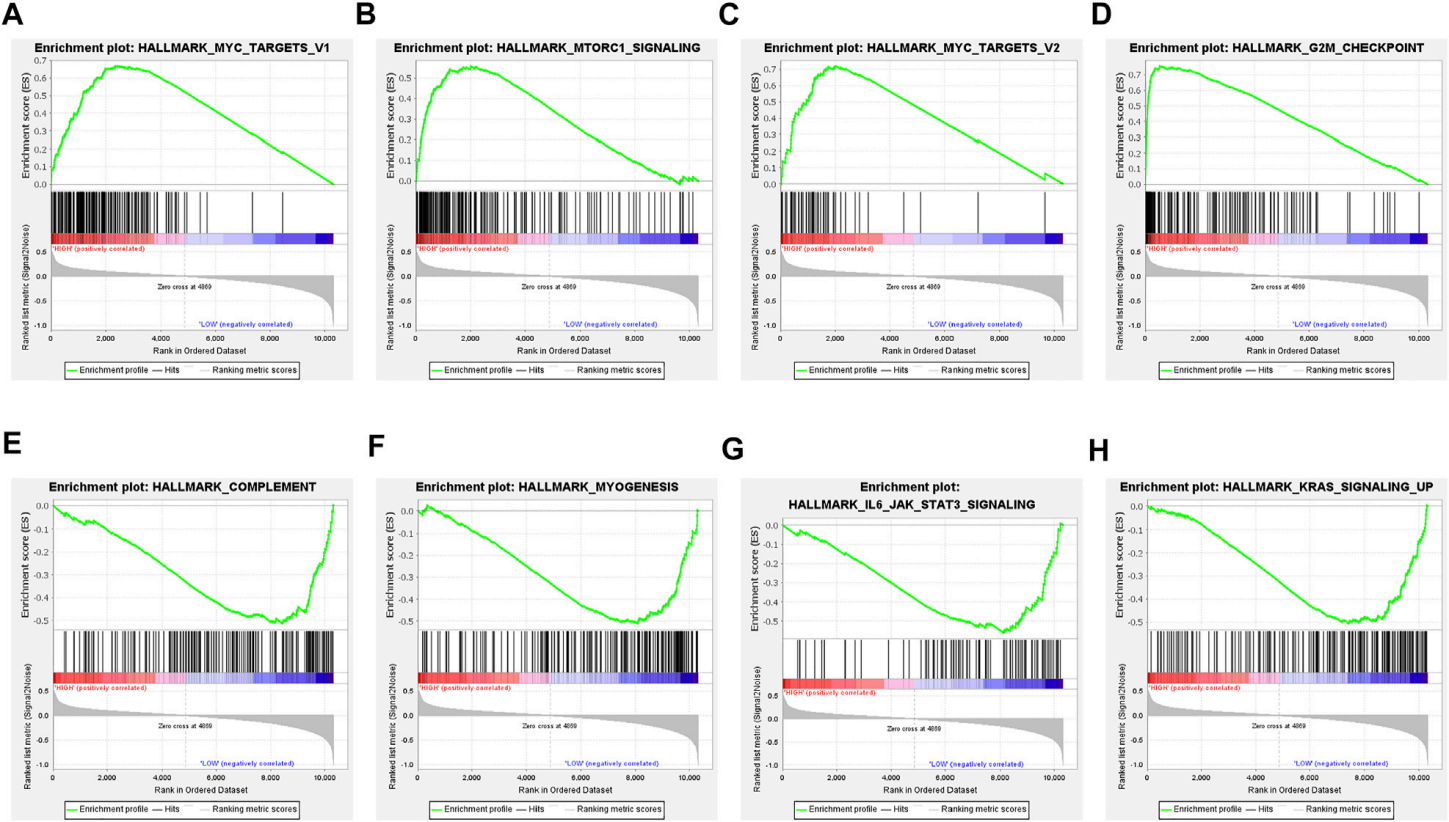
Gene set enrichment analysis (GSEA) in TMEscore groups. **(A**–**D)** Enrichment plots showing MYC targets V1, MTORC1 signaling, MYC targets V2, and G2M checkpoint in the high-TMEscore group. **(E**–**H)** Enrichment plots showing complement, myogenesis, IL6/JAK/STAT3 signaling, and KRAS signaling up in the low-TMEscore group.

For a comprehensive analysis of the potential regulatory mechanisms resulting in different TMEscore groups, we performed GSEA analysis between high and low TMEscore subgroups. The results showed that 23 pathways were enriched in different subgroups with FDR<0.25 (Supplementary Table S6). In high TMEscore group, MYC targets V1 (NES = 2.28 and FDR = 0.001), MTORC1 signaling (NES = 2.08 and FDR = 0.011), MYC targets V2 (NES = 2.08 and FDR = 0.012), G2M checkpoint (NES = 2.00 and FDR = 0.021), glycolysis (NES = 1.79 and FDR = 0.014), and other pathways were enriched (Figures 4A–D). Meanwhile, the results revealed that complement (NES = −1.74 and FDR = 0.231), inflammatory response (NES = −1.69 and FDR = 0.212), IL6/JAK/STAT3 signaling (NES = −1.64 and FDR = 0.181), IL2/STAT5 signaling up (NES = −1.58 and FDR = 0.212) and interferon gamma response (NES = −1.42 and FDR = 0.223), and other pathways were correlated with the low TMEscore (Figures 4E–H). It is suggested that the gene sets of the TMEscore high samples were enriched in cancer and tumor metabolism-related pathways, while the gene sets of the TMEscore low samples were enriched in DNA repair and immune response-related pathways.

### The Molecular Characteristics of Distinct TMEscore Subgroups

**FIGURE 5:**
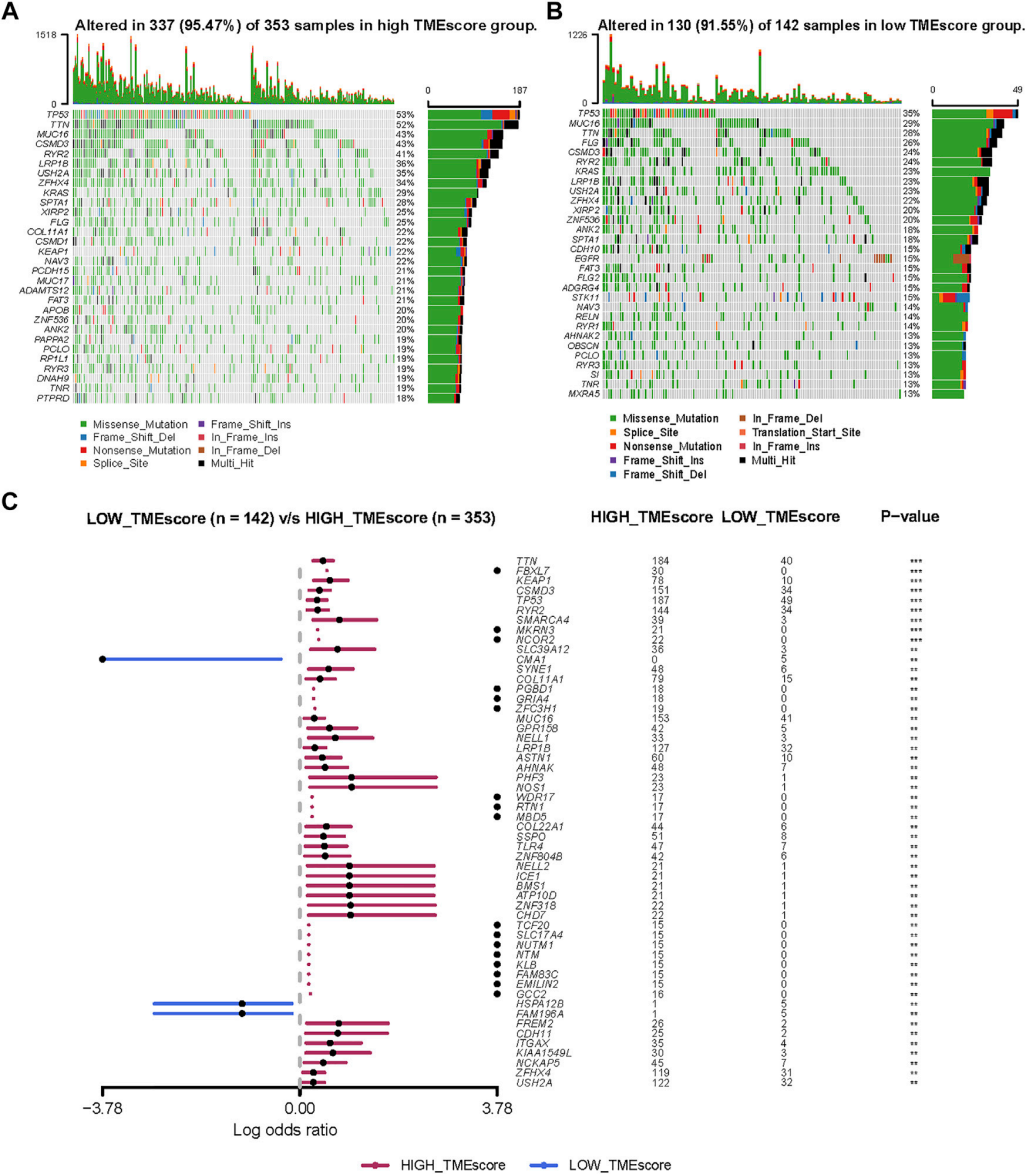
Molecular variations between low-TMEscore and high-TMEscore groups. **(A** and **B)** Mutation profiles of high-TMEscore and low-TMEscore groups. **(C)** Comparing differentially mutated genes between two subgroups by Fisher’s exact tests.

Genomic alterations and oncogenic signaling within the tumors have been reported to affect anti/pro-tumor immunity and TME activity (Hamarsheh et al., 2020; Kumagai et al., 2020; Zhou et al., 2020; Fountzilas et al., 2021), links between tumor mutations and TME subtypes needed to be investigated. To illustrate the somatic variants and acquire further biological insights into the immunological characteristics of LUAD between TMEscore subgroups, we utilized the Mutation Annotation Format (MAF) files and performed the variants annotation. We found higher mutation counts in the TME-high subgroup than in the TME-low subgroup. Missense variations were the most common mutation subtype, followed by nonsense and frameshift deletions. The oncoplot of tumor somatic mutation in the TCGA-LUAD cohort showed that TP53, TTN, and MUC16 gene mutations in the high-TMEscore group were approximately 20% higher than those in the low TMEscore group (Figures 5A and B). Among a total of 54 differential mutated genes between two groups (*p* < 0.01; Figure 5C), CMA1, HSPA12B, and FAM196A showed a higher mutation frequency in the low-TMEscore group. The other genes, such as TP53, TTN, and FBXL7, had a higher mutation frequency in the high-TMEscore group. Collectively, this analysis indicated that transcriptomic-based TME classification coupled with genomics analysis can be exploited for further studies.

### Combinations of TMEscore, Immune Checkpoints, and TMB Improve Risk Stratification and Survival Prediction

**FIGURE 6:**
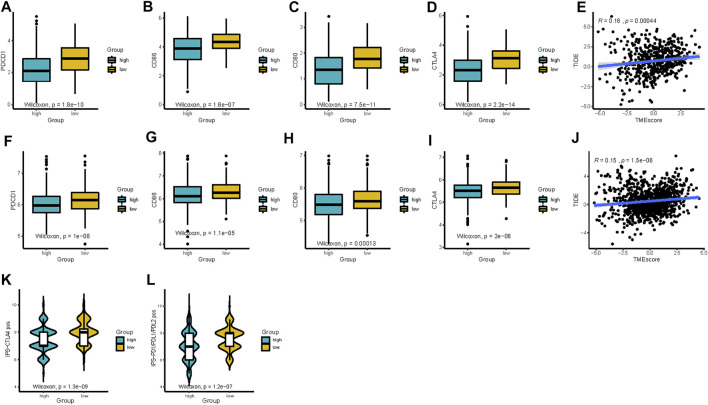
TMEscore in the prediction of immunotherapeutic benefits. **(A**–**D)** Expression of immune-checkpoint-relevant genes (PDCD1, CD86, CD80, and CTLA4) between high- and low-TMEscore groups in TCGA and **(F**–**I)** all 1,018 patients in GEO datasets after batch correction. **(E** and **J)** Relationships between TIDE and TMEscore in TCGA and GEO datasets. **(K** and **L)** Relative probabilities of response to anti-CTLA-4 and anti-PD-1/PD-L1 treatment (IPS score) in the low-TMEscore and high-TMEscore groups in TCGA cohort.

Previous studies have emphasized the importance of immune checkpoint genes in modulating immune infiltration (Keir et al., 2008; Andrews et al., 2019). Thus, we first compared the expression pattern of immune checkpoint genes between different patient groups delaminated by the TMEscore in TCGA-LUAD and GEO datasets. PDCD1, CD86, CD80, and CTLA4 showed significantly high expression in the low-TMEscore group than in the high-TMEscore group (Figures 6A–D, F–I), which was further confirmed in five independent validating cohorts (Supplementary Figure S4).

**FIGURE 7:**
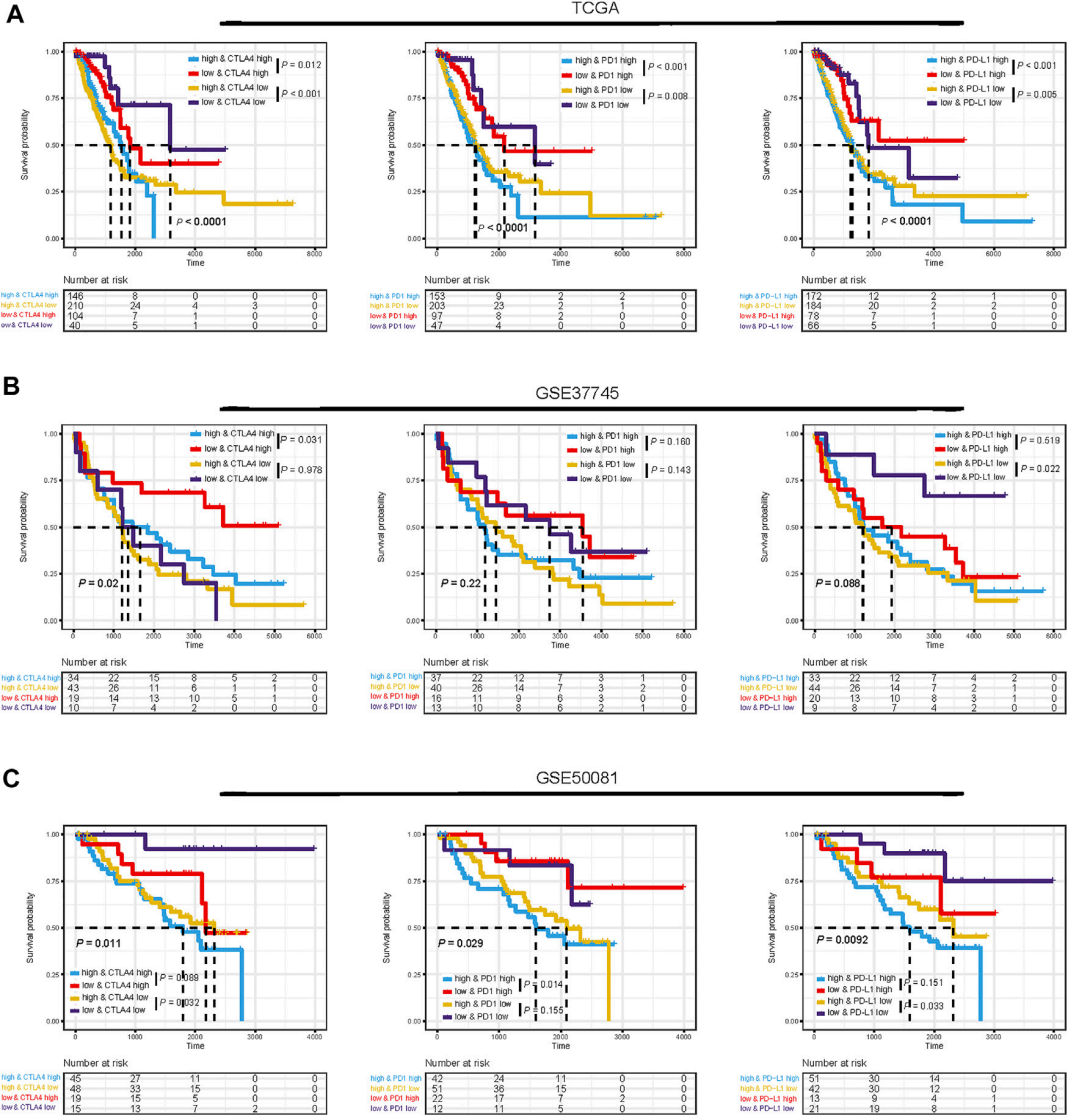
Impact of immune checkpoint gene expressions and TMEscore on clinical outcome. Kaplan–Meier survival curves of overall survival among four patient groups stratified by TMEscore and immune checkpoint genes (PD1, PD-L1, and CTLA-4) in TCGA dataset **(A)**, GSE37745 dataset **(B)**, and GSE50081 dataset **(C)**.

**FIGURE 8:**
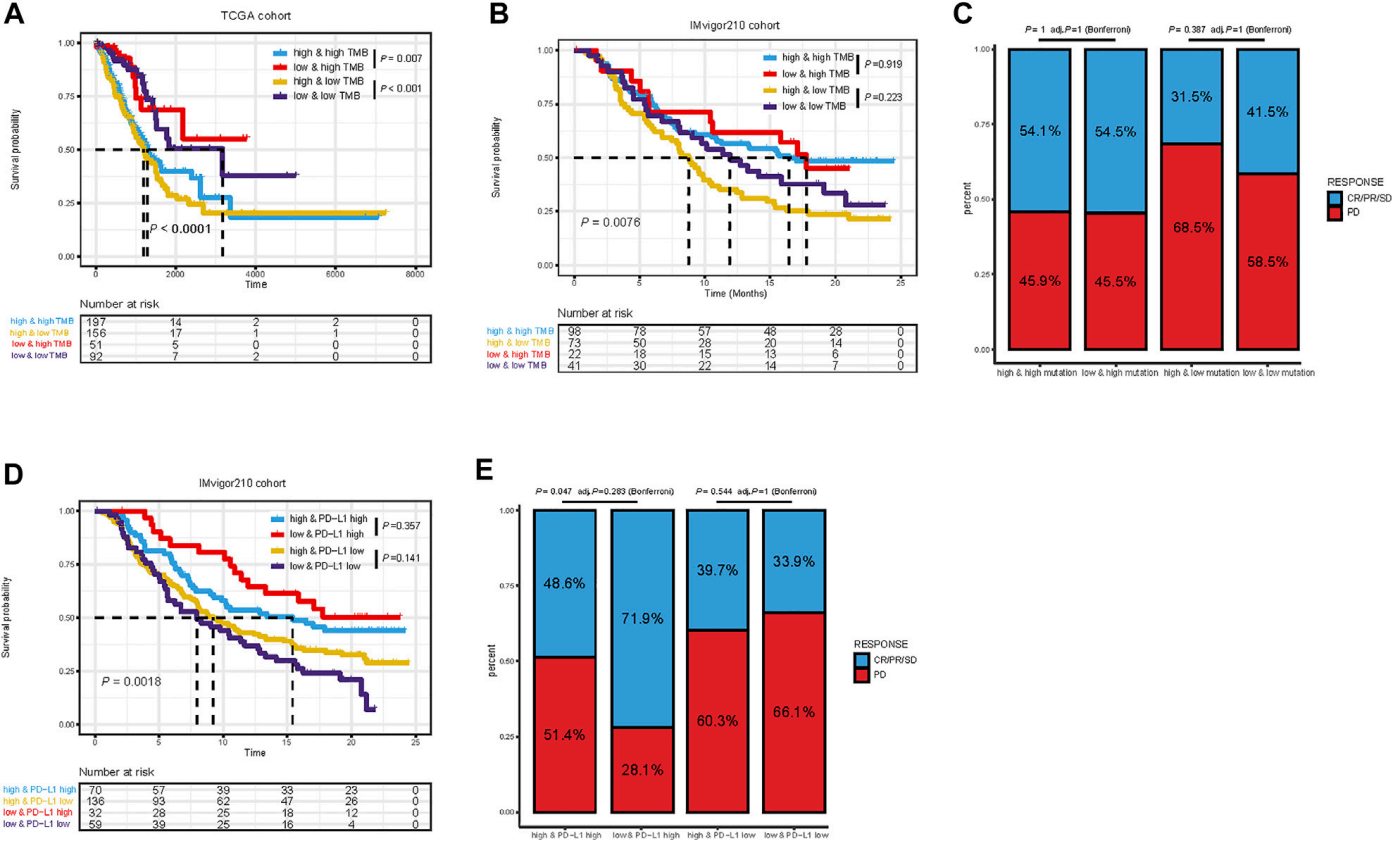
Kaplan–Meier survival curves of overall survival among four patient groups stratified by TMEscore and TMB in TCGA dataset **(A)** and IMvigor210 cohort **(B)**. Proportional representation of the objective response rate among subgroups categorized by TMEscore and TMB in the IMvigor210 cohort **(C)**. Kaplan–Meier survival curves of overall survival among four patient groups stratified by TMEscore and PD-L1 in the IMvigor210 cohort. **(D)** Proportional representation of the objective response rate among subgroups categorized by TMEscore and PD-L1 in the IMvigor210 cohort **(E)**. CR, complete response; PR, partial response; SD, stable disease; PD, progressive disease.

Considering the correlations between immune checkpoint genes and TMEscore, we next combined TMEscore with immune checkpoints expression to test whether they have an influence on OS in LUAD patients. Though survival analyses among four subgroups stratified by TMEscore and immune checkpoint gene expression, we displayed that patients with low PD-L1 and low TMEscore have prolonged OS compared to those with low PD-L1 and high TMEscore (*p* = 0.005), and among patients with high PD-L1 expression, a lower TMEscore signified a remarkably better survival (*p* < 0.001) (Figure 7A). We also found similar survival patterns among four patient subgroups stratified by TMEscore and PD1/CTLA-4 expressions in the TCGA cohort (Figure 7A). We then confirmed the results in the other five validation cohorts (Figures 7B,C and Supplementary Figure S5). In concordance with the TCGA dataset, patients with low TMEscore have significantly better survival relative to the high TMEscore group, even though with similar expression levels of immune checkpoint genes (Figures 7B,C and Supplementary Figure S5). In addition, TMB has been shown to have the potential to generate a larger number of neoantigens and make them more immunogenic (Schumacher and Schreiber, 2015), which is strongly associated with clinical outcomes and response of immune checkpoint blockade response (Yarchoan et al., 2017; Chan et al., 2019). We found that patients with low TMB and high TMEscore had the worst prognosis (Figure 8A). It is suggested that TMEscore, immune checkpoint genes, and TMB can complement each other as prognostic biomarkers.

### The TMEscore Predicts Clinical Outcomes of Immunotherapy

Given the linkage between TMEscore and immune checkpoint genes as well as TMB, we further explore the predictive potential of TMEscore for immune checkpoint blockade response through analyzing the correlation of TMEscore and published immunotherapy predictors, including TIDE and IPS. The relationship between TIDE and TMEscore was investigated in TCGA and GEO datasets. As expected, the high-TMEscore group was characterized by a significantly higher TIDE score (Figures 6E and J). The IPS values (IPS-PD-1/PD-L1/PD-L2_pos and IPS-CTLA-4_pos) increased in the low-TMEscore group compared to the high-TMEscore group in TCGA (Figures 6K and L). It is likely that the patients in the low-TMEscore group may have a better immune microenvironment and respond better to ICIs than those in the high-TMEscore group.

Furthermore, the practicability of the TMEscore was further evaluated for speculation of the therapeutic benefit for ICI treated patients. The patients who received anti-PD-L1 immunotherapy in the IMvigor210 cohort were assigned based on high and low TME scores. Given the contraindicatory prognostic and predictive value of TMEscore, TMB, and immune checkpoint gene expression (PD-L1, PD-1, and CTLA4), we next evaluated the synergistic effect of these biomarkers in the prognostic and predictive stratification of LUAD. Consistent with previous results, stratified survival analysis revealed that the TMB status did not interfere with TMEscore-based predictions. Subtypes of the combination of TMEscore and TMB showed significant survival differences (log-rank test, *p* = 0.0076; Figure 8B). On the other hand, Kaplan–Meier analysis revealed patients in the IMvigor210 cohort with TMEscore low and PD-L1 high obtained most favorable OS than either single positive (TME low or PD-L1 high) or dual negative (TMEscore high PD-L1 low, *p* = 0.0018, Figure 8D). In addition, analysis of objective response also supported that TMEscore low and PD-L1 high subgroup represented an increased proportion of PR/CR/SD than either single positive (TMEscore low or PD-L1 high) or dual negative (*p* = 0.047, Figure 8E). Taken together, these findings indicate the TME classification system and scoring system may explain the effectiveness of immunotherapy in patients with low TMB and low PD-L1, and these distinct classification systems, TMEscore, PD-L1, and TMB might function as complementary factors for the prediction of immunotherapy.

## Discussion

Although immune checkpoint inhibitors (ICIs) have revolutionized treatment strategies of lung cancer, the overall response rate of ICI monotherapies is still limited and no more than 20% in NSCLC patients with EGFR/ALK wild-type (Doroshow et al., 2019). It has been reported that TME plays a crucial role in cancer development and anti-tumor process, especially the immunotherapy response in cancers (Lu et al., 2020; Ye et al., 2022). Therefore, characterizing the tumor–immune microenvironment can improve the personalized immunotherapeutic strategies.

Multi-omics data are often used for generating various predictive or prognostic models through machine learning or statistical modeling methods (Xu et al., 2021). However, to date, comprehensive analyses based on integrated genomic and transcriptomic profiles of the tumor and its TME remain rare and lack efficient and useful models. Therefore, we constructed a scoring system to classify and quantify the comprehensive tumor immune landscape based on an immune-cell phenotype algorithm and validation in external LUAD cohorts.

Transcriptomic analysis offers an opportunity to dissect the complexity of tumors, including TME, dynamically regulating cancer progression and influencing therapeutic outcomes (Cieslik and Chinnaiyan, 2018; Thorsson et al., 2018). In our study, we identified four distinct immune subtypes characterized by different biological processes and prognosis, using “CIBERSORT” algorithm to analyze the transcriptomic data of TCGA-LUAD samples. Furthermore, we established the TME scores to characterize and quantify the immune infiltration patterns for each LUAD patient based on the DEGs among the distinct subtypes. The TMEscore displayed as a reliable prognostic immune-related biomarker for LUAD, with worse survival in TMEscore-high patients and better survival in TMEscore-low patients and in both TCGA and other five independent GEO cohorts. In addition, enriched pathways and genomic alterations were also analyzed and compared in different TMEscore subgroups, and we observed that a high TMEscore was significantly correlated with more aggressive molecular changes such as TP53 mutations. As expected from its increased immune gene expression, the TMEscore-low subtype showed overexpression of PD-L1, PD-1, CTLA4, and associations of other markers of sensitivity to immunotherapy, including IPS score and TIDE score. Our findings also revealed that the TMEscore is a robust and reliable prognostic tool and predictive indicator of the response to immunotherapy in the IMvigor210 cohort. With further in-depth investigation, our TMEscore might be utilized as an important supplementary predictor to LUAD immunotherapy.

The tumor microenvironment (TME) is a complex interface between cancer cells, stroma, and infiltrating immune cells (Fridman et al., 2012). A previous study demonstrated that the tumor microenvironment contexture plays a key role in tumor development and immunotherapeutic efficacy (Stankovic et al., 2018). TME heterogeneity, which impacts tumor progression and prognosis, has been identified in cancers, especially LUAD (Lavin et al., 2017; Zhang et al., 2019; Chen et al., 2020; Nguyen et al., 2021). In addition, the difference in TME patterns was found to be correlated to tumor heterogeneity and treatment diversity (Jia et al., 2018; Vitale et al., 2021). Considering the individual heterogeneity of the immune milieu, it was demanded to quantify the TME patterns of individual tumors. Here, using the “CIBERSORT” algorithm, we identified 22 human immune-cell phenotypes and generated an individualized TMEscore to assess TME patterns. Our study represents an essential step toward understanding the crosstalk between malignant cells and immune cells in LUAD.

Our findings of the TCGA molecular mutations displayed significant differences in distributions across the TMEscore subgroups. The largest difference in mutations between subgroups was in TP53 mutations, which were more common in TME-high samples than in TME-low samples (53 vs. 35%). TP53 mutation is not only the most common genetic event in NSCLC but also reported to be associated with poor prognosis in cancers, especially non-small cell lung cancer (Ozaki and Nakagawara, 2011). TP53 mutation could affect disease progression, tumor cell characteristics, and the therapeutic effect of different therapeutics (Wu and Hwang, 2019). In addition, the more enrichment of KEAP1 mutations in TMEscore high tumors than TMEscore low may be one potential explanation for the distinct performance of ICI efficacy in LUAD. KEAP1 mutations were reported to be enriched in patients with high TMB lacking T-cell infiltration and immunologically cold (Marinelli et al., 2020), which have been associated with decreased efficacy of ICIs in NSCLC in published studies (Papillon-Cavanagh et al., 2020; Di Federico et al., 2021). The differences in their molecular characteristics between TMEscore subgroups might contribute to the diverse immunogenic features and consequently varied responses to immunotherapy.

To acquire a deeper insight into the biological feature of the TMEscore subgroups, we further investigated enriched pathways and immune characteristics of different TMEscore subgroups. Patients with the low-TMEscore subtype, whose molecular traits, including an abundance of infiltration immune active cells, enhanced enrichment of immune-related pathways, such as interferon gamma response, complement, inflammatory response, were previously reported to predict the efficacy of pembrolizumab. In addition, we also observed elevated IL6/JAK/STAT3 signaling pathway in the low-TMEscore group, modulating the IFN-γ-induced expression of PD-L1 (Zhang et al., 2021). Collectively, the designated distinct TMEscore subtypes of LUAD were identified, and the crucial insights into the immunologic features of these subtypes were provided. Meanwhile, we proved that the TMEscore showed significant correlations with immune checkpoint genes (PD-L1, PD-1, and CTLA-4), TMB, and other biomarkers of immunotherapy, including IPS and TIDE, indicating that TMEscore possessed the potential to predict the response to immunotherapy. Previous studies have demonstrated that some biomarkers, such as TIDE and IPS, could predict patient response to immunotherapy. TIDE, a creative computational method to identify the induction of T-cell dysfunction in tumors with high infiltration of cytotoxic T lymphocytes and the prevention of T-cell infiltration in tumors with low-CTL levels (Jiang et al., 2018), has been proven to predict the outcomes of cancers treated with ICIs (Jiang et al., 2018). In addition, IPS was developed to quantitatively predict patients’ response to anti-PD-1/PD-L1 and anti-CTLA-4 therapies based on an 18-gene signature including genes that reflect an ongoing adaptive Th1 and cytotoxic CD8 T-cell response (Charoentong et al., 2017). Thus, the low-TMEscore patients presenting high IPS and low TIDE scores may have a better response to immunotherapy. However, both TIDE and IPS focused on the function and status of T cells, which could not fully reflect the complexity of the TME involved in the response to immunotherapy. Therefore, our scoring system exhibits promising clinical flexibility for the predictive value of anti-PD-(L)1 therapy.

Furthermore, patients in the high-risk subgroup presented with a higher level of immune checkpoint molecules and showed higher immunogenicity. However, PD-L1 expression and TMB are neither the only nor the satisfying tool to identify NSCLC patients that might benefit from therapy with immune checkpoints inhibitors (Klein et al., 2021). One critical obstacle impeding the extensive utility of TMB and PD-L1 expression is the determination of feasible cut-off values. Moreover, these two predictors only focused on the intrinsic features of tumors and may not cover other situations involved in antitumor immune responses such as TME. Notably, we demonstrated that it is reasonable to combine TMEscore with PD-L1 or TMB together, and thus it might help make clinical decisions in LUAD. Patients with TMEscore-low PD-L1 high or TMB high should be preferentially recommended for ICI treatment, while patients with TMEscore-low PD-L1 low, or TMEscore-high PD-L1 high can optionally consider anti-PD- (L)1 therapy; however, patients with TMEscore high/low PD-L1 or low TMB should carefully choose anti-PD-(L)1 therapy. Taking this step further, it is suggested that TMEscore can identify either potential sensitive patients with low PD-L1 expression/low TMB who may benefit or patients who do not respond to ICIs despite having a high PD-L1 expression/high TMB. In addition, we also explored the stability of our TMEscore model. We found that patients with a lower TMEscore were more likely to respond to ICB and had improved overall survival in the IMvigor210 cohort treated with checkpoint blockade. Collectively, combinations of TMEscore, TMB, and PD-L1 could be applied not only as refined prognostic stratification tools but also as more reliable predictive biomarkers for personalized immunotherapy treatment.

Our study provides a translational rationale for evaluating TME based on transcriptomic data and TMEscore as a biomarker for immunotherapy response in patients with LUAD. However, this study still has several limitations. First, while the composition of the TME has been recognized as a determinant of cancer progression and response to therapy, most analyses have focused on a limited proportion of cell types. Nonetheless, there are still numerous cellular and molecular mechanisms involved in immunotherapy, and our TMEscore may not cover the possible intra/extracellular situations involved in antitumor immune responses. Second, since this study was a retrospective analysis, the ability of the TMEscore in predicting survival and response to immunotherapy should be validated in a large-cohort, multi-center, and prospective study in the future. Third, all quantifications of gene expression are relative values, which makes it difficult to determine the absolute threshold and cut-off values for clinical application. Therefore, quantitative determinations of gene expression are also needed. Specifically, the underlying molecular mechanisms remain to be elucidated in LUAD *in vivo and in vitro*.

## Conclusion

In conclusion, our translational rationale for TME classification may help in distinguishing immune and molecular characteristics and predicting clinical outcomes of LUAD patients. These findings will further improve the implementation and utility of precisely personalized immunotherapeutic strategies in LUAD.

## Data Availability

The original contributions presented in the study are included in the article/Supplementary Material, further inquiries can be directed to the corresponding authors.

## Abbreviations

CI, confidence interval; DEGs, differential expression genes; ESTIMATE, Estimation of Stromal and Immune cells in Malignant Tumor tissues using Expression data; FDR, false discovery rate; GEO, Gene Expression Omnibus; GO, Gene Ontology; GSEA, gene set enrichment analysis; HR, hazard ratio; ICIs, immune checkpoint inhibitors; IPS, immunophenoscore; KEGG, Kyoto Encyclopedia of Genes and Genomes; LUAD, lung adenocarcinoma; PCA, principal component analysis; RNA-seq, RNA sequencing; TCGA, The Cancer Genome Atlas; TIDE, Tumor Immune Dysfunction and Exclusion; TIICs, tumor-infiltrating immune cells; TMB, tumor mutation burden; TME, tumor microenvironment.

## References

[B1] Andrews L. P. Yano H. Vignali D. A. A. (2019). Inhibitory Receptors and Ligands beyond PD-1, PD-L1 and CTLA-4: Breakthroughs or Backups. Nat. Immunol. 20, 1425–1434. 10.1038/s41590-019-0512-0 31611702

[B2] Belli C. Trapani D. Viale G. D'Amico P. Duso B. A. Della Vigna P. (2018). Targeting the Microenvironment in Solid Tumors. Cancer Treat. Rev. 65, 22–32. 10.1016/j.ctrv.2018.02.004 29502037

[B3] Chan T. A. Yarchoan M. Jaffee E. Swanton C. Quezada S. A. Stenzinger A. (2019). Development of Tumor Mutation burden as an Immunotherapy Biomarker: Utility for the Oncology Clinic. Ann. Oncol. 30, 44–56. 10.1093/annonc/mdy495 30395155 PMC6336005

[B4] Charoentong P. Finotello F. Angelova M. Mayer C. Efremova M. Rieder D. (2017). Pan-cancer Immunogenomic Analyses Reveal Genotype-Immunophenotype Relationships and Predictors of Response to Checkpoint Blockade. Cel Rep. 18, 248–262. 10.1016/j.celrep.2016.12.019 28052254

[B5] Chen N. Fang W. Zhan J. Hong S. Tang Y. Kang S. (2015). Upregulation of PD-L1 by EGFR Activation Mediates the Immune Escape in EGFR-Driven NSCLC: Implication for Optional Immune Targeted Therapy for NSCLC Patients with EGFR Mutation. J. Thorac. Oncol. 10, 910–923. 10.1097/jto.0000000000000500 25658629

[B6] Chen Y. P. Yin J. H. Li W. F. Li H. J. Chen D. P. Zhang C. J. (2020). Single-cell Transcriptomics Reveals Regulators Underlying Immune Cell Diversity and Immune Subtypes Associated with Prognosis in Nasopharyngeal Carcinoma. Cell Res. 30, 1024–1042. 10.1038/s41422-020-0374-x 32686767 PMC7784929

[B7] Cieslik M. Chinnaiyan A. M. (2018). Cancer Transcriptome Profiling at the Juncture of Clinical Translation. Nat. Rev. Genet. 19, 93–109. 10.1038/nrg.2017.96 29279605

[B8] Dejima H. Hu X. Chen R. Zhang J. Fujimoto J. Parra E. R. (2021). Immune Evolution from Preneoplasia to Invasive Lung Adenocarcinomas and Underlying Molecular Features. Nat. Commun. 12, 2722. 10.1038/s41467-021-22890-x 33976164 PMC8113327

[B9] Dempke W. C. M. Fenchel K. Dale S. P. (2018). Programmed Cell Death Ligand-1 (PD-L1) as a Biomarker for Non-small Cell Lung Cancer (NSCLC) Treatment-Are We Barking up the Wrong Tree? Transl. Lung Cancer Res. 7, S275–S279. 10.21037/tlcr.2018.04.18 30393621 PMC6193918

[B10] Di Federico A. De Giglio A. Parisi C. Gelsomino F. (2021). STK11/LKB1 and KEAP1 Mutations in Non-small Cell Lung Cancer: Prognostic rather than Predictive? Eur. J. Cancer 157, 108–113. 10.1016/j.ejca.2021.08.011 34500370

[B11] Doroshow D. B. Sanmamed M. F. Hastings K. Politi K. Rimm D. L. Chen L. (2019). Immunotherapy in Non-small Cell Lung Cancer: Facts and Hopes. Clin. Cancer Res. 25, 4592–4602. 10.1158/1078-0432.ccr-18-1538 30824587 PMC6679805

[B12] Fountzilas E. Kurzrock R. Hiep Vo H. Tsimberidou A. M. (2021). Wedding of Molecular Alterations and Immune Checkpoint Blockade: Genomics as a Matchmaker. J. Natl. Cancer Inst. 113, 1634–1647. 10.1093/jnci/djab067 PMC9890928 33823006

[B13] Fridman W. H. Pages F. Sautes-Fridman C. Galon J. (2012). The Immune Contexture in Human Tumours: Impact on Clinical Outcome. Nat. Rev. Cancer 12, 298–306. 10.1038/nrc3245 22419253

[B14] Gu C. Shi X. Dai C. Shen F. Rocco G. Chen J. (2020). RNA M(6)A Modification in Cancers: Molecular Mechanisms and Potential Clinical Applications. Innovation (N Y) 1, 100066. 10.1016/j.xinn.2020.100066 PMC8454620 34557726

[B15] Hamarsheh S. Gross O. Brummer T. Zeiser R. (2020). Immune Modulatory Effects of Oncogenic KRAS in Cancer. Nat. Commun. 11, 5439. 10.1038/s41467-020-19288-6 33116132 PMC7595113

[B16] Hinshaw D. C. Shevde L. A. (2019). The Tumor Microenvironment Innately Modulates Cancer Progression. Cancer Res. 79, 4557–4566. 10.1158/0008-5472.can-18-3962 31350295 PMC6744958

[B17] Jia Q. Wu W. Wang Y. Alexander P. B. Sun C. Gong Z. (2018). Local Mutational Diversity Drives Intratumoral Immune Heterogeneity in Non-small Cell Lung Cancer. Nat. Commun. 9, 5361. 10.1038/s41467-018-07767-w 30560866 PMC6299138

[B18] Jiang P. Gu S. Pan D. Fu J. Sahu A. Hu X. (2018). Signatures of T Cell Dysfunction and Exclusion Predict Cancer Immunotherapy Response. Nat. Med. 24, 1550–1558. 10.1038/s41591-018-0136-1 30127393 PMC6487502

[B19] Jiao D. Yang S. (2020). Overcoming Resistance to Drugs Targeting KRAS^G12C^ Mutation. Innovation (N Y) 1, 100035. 10.1016/j.xinn.2020.100035 PMC7491749 32939510

[B20] Kawai O. Ishii G. Kubota K. Murata Y. Naito Y. Mizuno T. (2008). Predominant Infiltration of Macrophages and CD8(+) T Cells in Cancer Nests Is a Significant Predictor of Survival in Stage IV Nonsmall Cell Lung Cancer. Cancer 113, 1387–1395. 10.1002/cncr.23712 18671239

[B21] Keir M. E. Butte M. J. Freeman G. J. Sharpe A. H. (2008). PD-1 and its Ligands in Tolerance and Immunity. Annu. Rev. Immunol. 26, 677–704. 10.1146/annurev.immunol.26.021607.090331 18173375 PMC10637733

[B22] Klein O. Kee D. Markman B. Carlino M. S. Underhill C. Palmer J. (2021). Evaluation of TMB as a Predictive Biomarker in Patients with Solid Cancers Treated with Anti-PD-1/CTLA-4 Combination Immunotherapy. Cancer Cell 39, 592–593. 10.1016/j.ccell.2021.04.005 33930312

[B23] Krishnamurthy N. Goodman A. M. Barkauskas D. A. Kurzrock R. (2021). STK11 Alterations in the Pan-Cancer Setting: Prognostic and Therapeutic Implications. Eur. J. Cancer 148, 215–229. 10.1016/j.ejca.2021.01.050 33744718 PMC10344467

[B24] Kumagai S. Togashi Y. Sakai C. Kawazoe A. Kawazu M. Ueno T. (2020). An Oncogenic Alteration Creates a Microenvironment that Promotes Tumor Progression by Conferring a Metabolic Advantage to Regulatory T Cells. Immunity 53, 187–203. 10.1016/j.immuni.2020.06.016 32640259

[B25] Lavin Y. Kobayashi S. Leader A. Amir E. D. Elefant N. Bigenwald C. (2017). Innate Immune Landscape in Early Lung Adenocarcinoma by Paired Single-Cell Analyses. Cell 169, 750–765. 10.1016/j.cell.2017.04.014 28475900 PMC5737939

[B26] Lee C. K. Man J. Lord S. Links M. Gebski V. Mok T. (2017). Checkpoint Inhibitors in Metastatic EGFR-Mutated Non-small Cell Lung Cancer-A Meta-Analysis. J. Thorac. Oncol. 12, 403–407. 10.1016/j.jtho.2016.10.007 27765535

[B27] Lu Z. Peng Z. Liu C. Wang Z. Wang Y. Jiao X. (2020). Current Status and Future Perspective of Immunotherapy in Gastrointestinal Cancers. Innovation (N Y) 1, 100041. 10.1016/j.xinn.2020.100041 PMC8454608 34557714

[B28] Mantovani A. Marchesi F. Malesci A. Laghi L. Allavena P. (2017). Tumour-associated Macrophages as Treatment Targets in Oncology. Nat. Rev. Clin. Oncol. 14, 399–416. 10.1038/nrclinonc.2016.217 28117416 PMC5480600

[B29] Marinelli D. Mazzotta M. Scalera S. Terrenato I. Sperati F. D'Ambrosio L. (2020). KEAP1-driven Co-mutations in Lung Adenocarcinoma Unresponsive to Immunotherapy Despite High Tumor Mutational burden. Ann. Oncol. 31, 1746–1754. 10.1016/j.annonc.2020.08.2105 32866624

[B30] Mazzaschi G. Leonetti A. Minari R. Gnetti L. Quaini F. Tiseo M. (2021). Modulating Tumor Microenvironment: A Review on STK11 Immune Properties and Predictive vs Prognostic Role for Non-small-cell Lung Cancer Immunotherapy. Curr. Treat. Options. Oncol. 22, 96. 10.1007/s11864-021-00891-8 34524570

[B31] Miller K. D. Nogueira L. Mariotto A. B. Rowland J. H. Yabroff K. R. Alfano C. M. (2019). Cancer Treatment and Survivorship Statistics, 2019. CA Cancer J. Clin. 69, 363–385. 10.3322/caac.21565 31184787

[B32] Newman A. M. Liu C. L. Green M. R. Gentles A. J. Feng W. Xu Y. (2015). Robust Enumeration of Cell Subsets from Tissue Expression Profiles. Nat. Methods 12, 453–457. 10.1038/nmeth.3337 25822800 PMC4739640

[B33] Nguyen P. H. D. Ma S. Phua C. Z. J. Kaya N. A. Lai H. L. H. Lim C. J. (2021). Intratumoural Immune Heterogeneity as a Hallmark of Tumour Evolution and Progression in Hepatocellular Carcinoma. Nat. Commun. 12, 227. 10.1038/s41467-020-20171-7 33431814 PMC7801667

[B34] Ostman A. (2012). The Tumor Microenvironment Controls Drug Sensitivity. Nat. Med. 18, 1332–1334. 10.1038/nm.2938 22961158

[B35] Ostrand-Rosenberg S. Fenselau C. (2018). Myeloid-Derived Suppressor Cells: Immune-Suppressive Cells that Impair Antitumor Immunity and Are Sculpted by Their Environment. J. Immunol. 200, 422–431. 10.4049/jimmunol.1701019 29311384 PMC5765878

[B36] Ozaki T. Nakagawara A. (2011). Role of P53 in Cell Death and Human Cancers. Cancers (Basel) 3, 994–1013. 10.3390/cancers3010994 24212651 PMC3756401

[B37] Papillon-Cavanagh S. Doshi P. Dobrin R. Szustakowski J. Walsh A. M. (2020). STK11 and KEAP1 Mutations as Prognostic Biomarkers in an Observational Real-World Lung Adenocarcinoma Cohort. ESMO Open 5, e000706. 10.1136/esmoopen-2020-000706 32312757 PMC7199918

[B38] Pardoll D. M. (2012). The Blockade of Immune Checkpoints in Cancer Immunotherapy. Nat. Rev. Cancer 12, 252–264. 10.1038/nrc3239 22437870 PMC4856023

[B39] Quail D. F. Joyce J. A. (2013). Microenvironmental Regulation of Tumor Progression and Metastasis. Nat. Med. 19, 1423–1437. 10.1038/nm.3394 24202395 PMC3954707

[B40] Schumacher T. N. Schreiber R. D. (2015). Neoantigens in Cancer Immunotherapy. Science 348, 69–74. 10.1126/science.aaa4971 25838375

[B41] Shimizu K. Nakata M. Hirami Y. Yukawa T. Maeda A. Tanemoto K. (2010). Tumor-infiltrating Foxp3+ Regulatory T Cells are Correlated with Cyclooxygenase-2 Expression and Are Associated with Recurrence in Resected Non-small Cell Lung Cancer. J. Thorac. Oncol. 5, 585–590. 10.1097/jto.0b013e3181d60fd7 20234320

[B42] Siegel R. L. Miller K. D. Jemal A. (2019). Cancer Statistics, 2019. CA Cancer J. Clin. 69, 7–34. 10.3322/caac.21551 30620402

[B43] Sotiriou C. Wirapati P. Loi S. Harris A. Fox S. Smeds J. (2006). Gene Expression Profiling in Breast Cancer: Understanding the Molecular Basis of Histologic Grade to Improve Prognosis. J. Natl. Cancer Inst. 98, 262–272. 10.1093/jnci/djj052 16478745

[B44] Stankovic B. Bjorhovde H. A. K. Skarshaug R. Aamodt H. Frafjord A. Muller E. (2018). Immune Cell Composition in Human Non-small Cell Lung Cancer. Front. Immunol. 9, 3101. 10.3389/fimmu.2018.03101 30774636 PMC6367276

[B45] Subramanian A. Tamayo P. Mootha V. K. Mukherjee S. Ebert B. L. Gillette M. A. (2005). Gene Set Enrichment Analysis: a Knowledge-Based Approach for Interpreting Genome-wide Expression Profiles. Proc. Natl. Acad. Sci. 102, 15545–15550. 10.1073/pnas.0506580102 16199517 PMC1239896

[B46] Sun H. Liu S. Y. Zhou J. Y. Xu J. T. Zhang H. K. Yan H. H. (2020). Specific TP53 Subtype as Biomarker for Immune Checkpoint Inhibitors in Lung Adenocarcinoma. EBioMedicine 60, 102990. 10.1016/j.ebiom.2020.102990 32927274 PMC7494676

[B47] Sung H. Ferlay J. Siegel R. L. Laversanne M. Soerjomataram I. Jemal A. (2021). Global Cancer Statistics 2020: GLOBOCAN Estimates of Incidence and Mortality Worldwide for 36 Cancers in 185 Countries. CA Cancer J. Clin. 71, 209–249. 10.3322/caac.21660 33538338

[B48] Thorsson V. Gibbs D. L. Brown S. D. Wolf D. Bortone D. S. Ou Yang T. H. (2018). The Immune Landscape of Cancer. Immunity 48, 812–830. 10.1016/j.immuni.2018.03.023 29628290 PMC5982584

[B49] Topalian S. L. Drake C. G. Pardoll D. M. (2015). Immune Checkpoint Blockade: a Common Denominator Approach to Cancer Therapy. Cancer Cell 27, 450–461. 10.1016/j.ccell.2015.03.001 25858804 PMC4400238

[B50] Vitale I. Shema E. Loi S. Galluzzi L. (2021). Intratumoral Heterogeneity in Cancer Progression and Response to Immunotherapy. Nat. Med. 27, 212–224. 10.1038/s41591-021-01233-9 33574607

[B51] Wagner G. P. Kin K. Lynch V. J. (2012). Measurement of mRNA Abundance Using RNA-Seq Data: RPKM Measure Is Inconsistent Among Samples. Theor. biosciences 131, 281–285. 10.1007/s12064-012-0162-3 22872506

[B52] Wellenstein M. D. de Visser K. E. (2018). Cancer-Cell-Intrinsic Mechanisms Shaping the Tumor Immune Landscape. Immunity 48, 399–416. 10.1016/j.immuni.2018.03.004 29562192

[B53] Wu C. H. Hwang M. J. (2019). Risk Stratification for Lung Adenocarcinoma on EGFR and TP53 Mutation Status, Chemotherapy, and PD-L1 Immunotherapy. Cancer Med. 8, 5850–5861. 10.1002/cam4.2492 31407494 PMC6792489

[B54] Wu F. Fan J. He Y. Xiong A. Yu J. Li Y. (2021a). Single-cell Profiling of Tumor Heterogeneity and the Microenvironment in Advanced Non-small Cell Lung Cancer. Nat. Commun. 12, 2540. 10.1038/s41467-021-22801-0 33953163 PMC8100173

[B55] Wu T. Hu E. Xu S. Chen M. Guo P. Dai Z. (2021b). clusterProfiler 4.0: A Universal Enrichment Tool for Interpreting Omics Data. Innovation (N Y) 2, 100141. 10.1016/j.xinn.2021.100141 PMC8454663 34557778

[B56] Xu Y. Liu X. Cao X. Huang C. Liu E. Qian S. (2021). Artificial Intelligence: A Powerful Paradigm for Scientific Research. Innovation (N Y) 2, 100179. 10.1016/j.xinn.2021.100179 PMC8633405 34877560

[B57] Yarchoan M. Hopkins A. Jaffee E. M. (2017). Tumor Mutational Burden and Response Rate to PD-1 Inhibition. N. Engl. J. Med. 377, 2500–2501. 10.1056/nejmc1713444 29262275 PMC6549688

[B58] Ye Y. Zhang Y. Yang N. Gao Q. Ding X. Kuang X. (2022). Profiling of Immune Features to Predict Immunotherapy Efficacy. Innovation (N Y) 3, 100194. 10.1016/j.xinn.2021.100194 PMC8688727 34977836

[B59] Zhang Q. He Y. Luo N. Patel S. J. Han Y. Gao R. (2019). Landscape and Dynamics of Single Immune Cells in Hepatocellular Carcinoma. Cell 179, 829–845 e20. 10.1016/j.cell.2019.10.003 31675496

[B60] Zhang L. Kuca K. You L. Zhao Y. Musilek K. Nepovimova E. (2021). Signal Transducer and Activator of Transcription 3 Signaling in Tumor Immune Evasion. Pharmacol. Ther. 230, 107969. 10.1016/j.pharmthera.2021.107969 34450232

[B61] Zhou Y. Bian S. Zhou X. Cui Y. Wang W. Wen L. (2020). Single-Cell Multiomics Sequencing Reveals Prevalent Genomic Alterations in Tumor Stromal Cells of Human Colorectal Cancer. Cancer Cell 38, 818–828. 10.1016/j.ccell.2020.09.015 33096021

