# Supplementary material for: Characterization of the Immune Cell Infiltration Landscape Uncovers Prognostic and Immunogenic Characteristics in Lung Adenocarcinoma
Source: Front Genet. 2022 May 23;13:902577. doi: 10.3389/fgene.2022.902577 (PMC9168373; doi:10.3389/fgene.2022.902577)
Supplement: Supplementary file 1 [file DataSheet2.docx]

Supplementary Figure S1. Overview of study design.


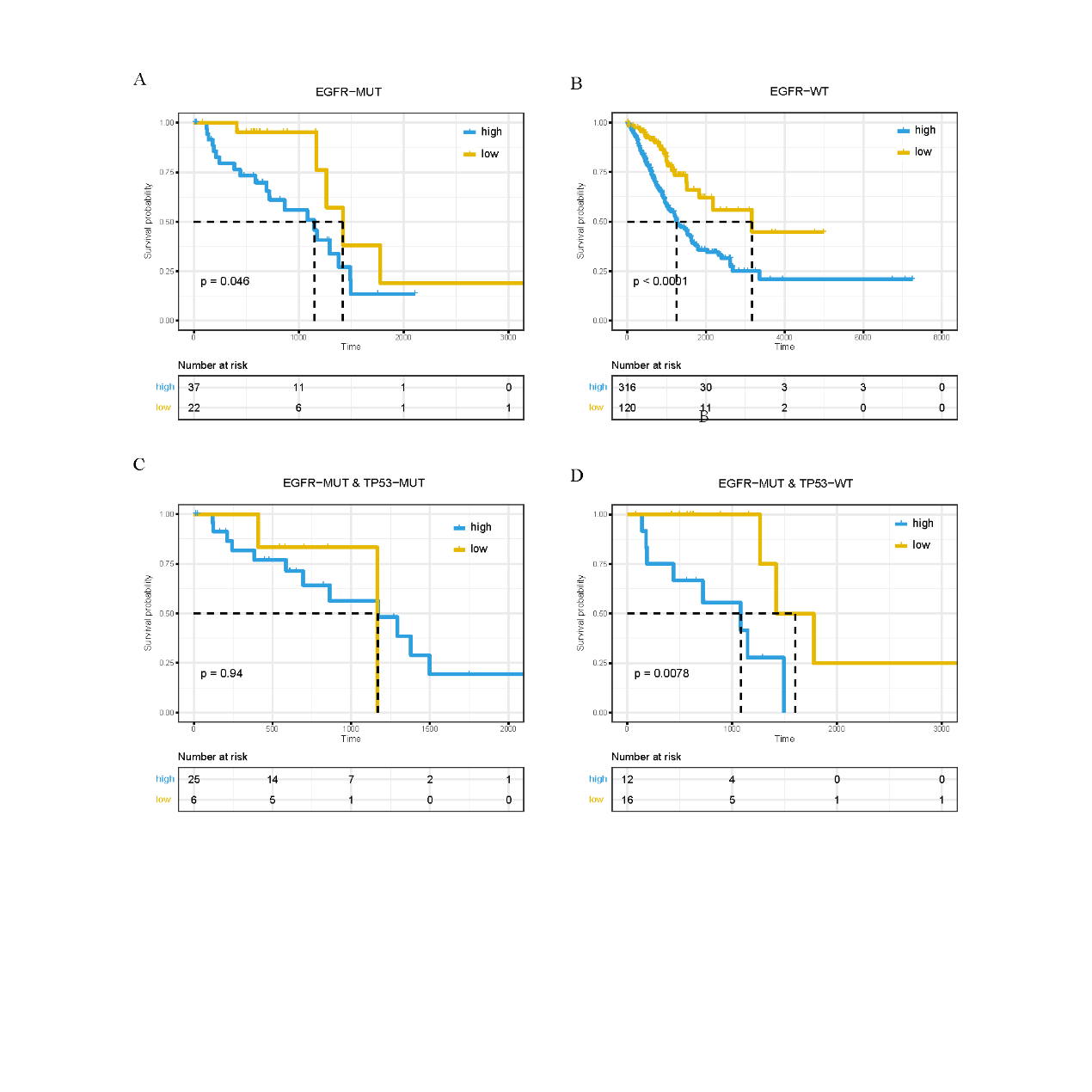


Supplementary Figure S2. The prognostic performance of TMEscore stratified by EGFR and TP53 mutation status. Kaplan-Meier curves of overall survival for high and low TMEscore in EGFR-MUT(EGFR-mutation) (A), EGFR-WT (EGFR-wild type) (B), EGFR-MUT& TP53-MUT (EGFR-mutation synchronized with TP53-mutation) (C), EGFR-MUT& TP53-WT (EGFR -mutation synchronized with TP53- wild type) (D) LUAD patients.


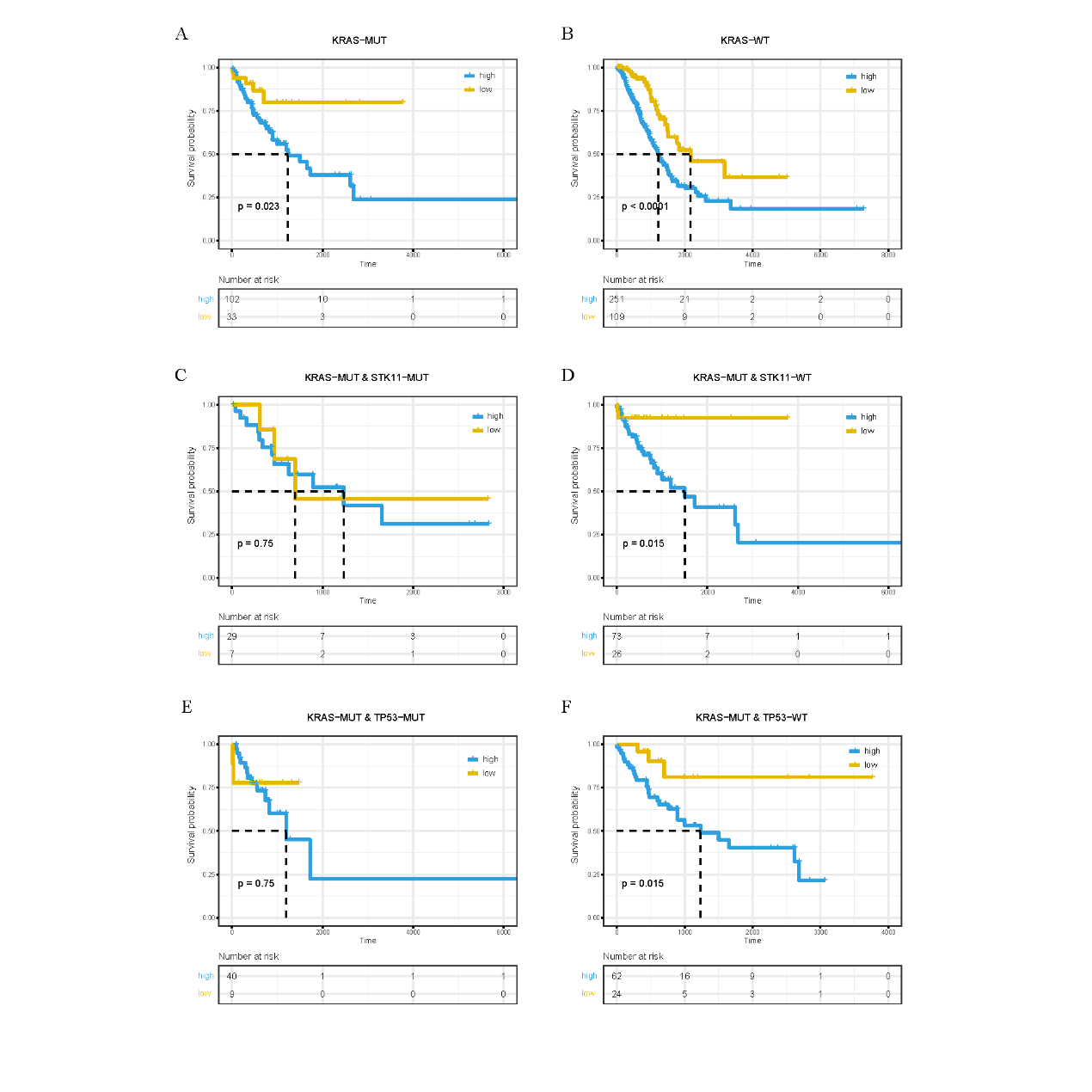


Supplementary Figure S3. The prognostic performance of TMEscore grouped by KRAS, STK-11 and TP53 mutation status. Kaplan-Meier curves of overall survival for high and low TMEscore in KRAS-MUT(KRAS-mutation) (A), KRAS-WT (KRAS-wild type) (B), KRAS-MUT& STK-11-MUT (KRAS-mutation synchronized with STK-11-mutation) (C), KRAS-MUT& STK-11-WT (KRAS-mutation synchronized with STK-11- wild type) (D), KRAS-MUT& TP53-MUT (KRAS-mutation synchronized with TP-53-mutation) (E), and KRAS-MUT& TP53-WT (KRAS-mutation synchronized with TP53- wild type) (F) LUAD patients.


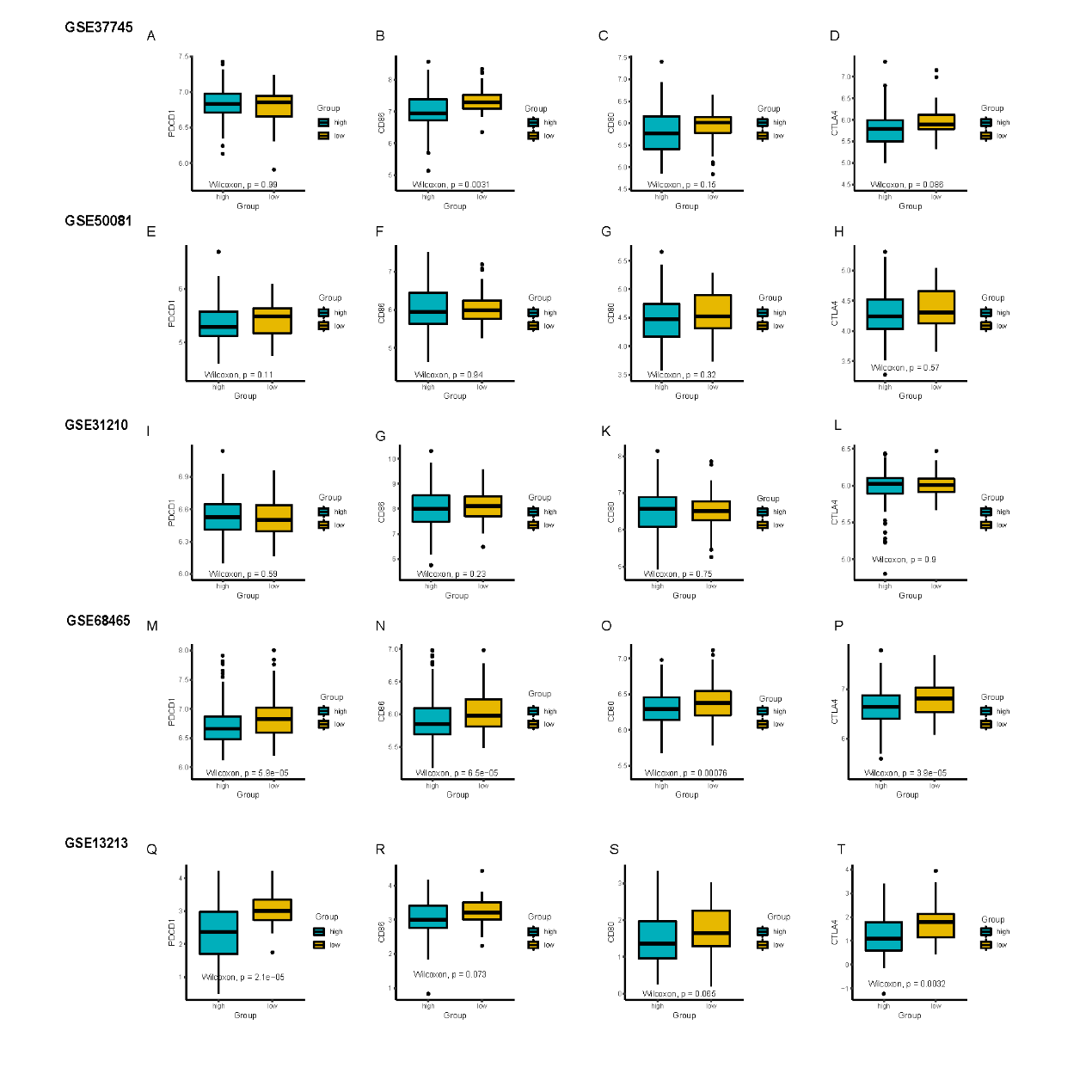


Supplementary Figure S4. Expression of immune-checkpoint-relevant genes between high and low TMEscore groups in five GEO datasets.


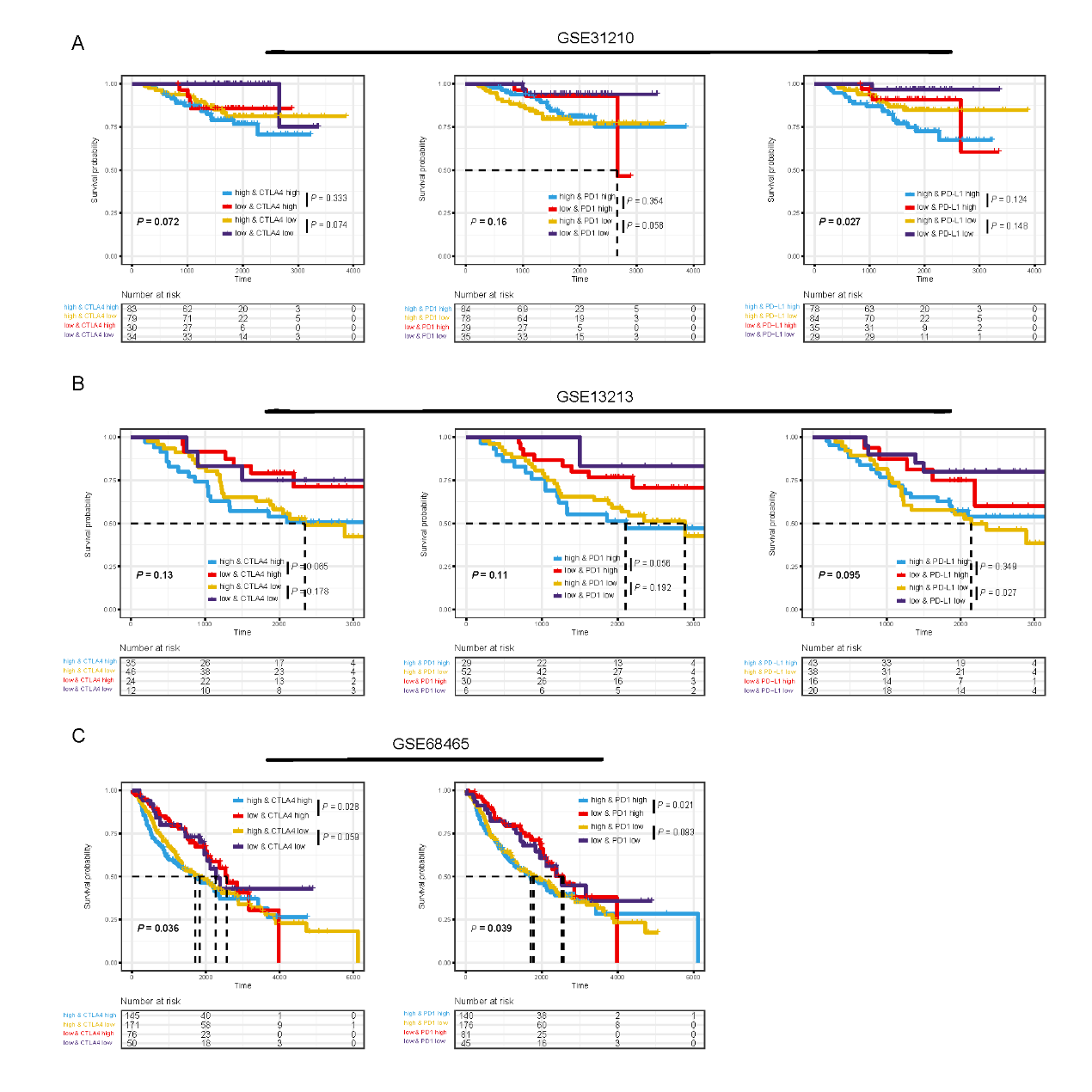


Supplementary Figure S5. Kaplan-Meier survival curves of overall survival among four patient groups stratified by TMEscore and immune checkpoint genes (PD1, PD-L1, and CTLA-4) in GSE31320 data set (A), GSE13213 data set (B) and GSE68465 data set (C).
